# A culturally integrated framework for infant nutrition beyond health education: lessons and a model from the Tibetan population

**DOI:** 10.3389/fpubh.2026.1848621

**Published:** 2026-07-23

**Authors:** Xi Huang, Yingxin Li, Wenqian Su, Yue Li, Yanling Hu, Qiong Chen

**Affiliations:** 1 Department of Neonatology Nursing, West China Second University Hospital, Sichuan University, Chengdu, China; 2 Key Laboratory of Birth Defects and Related Diseases of Women and Children, Sichuan University, Ministry of Education, Chengdu, China

**Keywords:** cultural adaptation, health equity, infant and young child feeding, intervention framework, malnutrition, socio-ecological model, Tibetan ethnicity

## Abstract

**Background:**

Improving infant and young child nutrition among marginalized ethnic groups urgently requires a paradigm shift from traditional health education to culturally integrated systemic interventions. Nutrition in the first 1,000 days is critical for long-term health and human capital development. Amid global efforts to eliminate health disparities, Tibetan infants face severe nutritional challenges, with stunting and anemia rates far exceeding national averages. Feeding practices, rooted in their unique high-altitude environment and deep cultural traditions, are a key social determinant of their health.

**Objective:**

This study aimed to comprehensively describe the traditional feeding practices among Tibetan infants aged 0–24 months via an integrative review, assess their health impacts, analyze influencing factors using the socio-ecological model, and ultimately construct a multi-level, culturally adapted public health intervention framework.

**Methods:**

An integrative review methodology was employed. Multiple English and Chinese databases (PubMed, Web of Science, Scopus, CNKI, etc.) were systematically searched for quantitative, qualitative, and mixed-methods studies on Tibetan infant feeding practices. Evidence was thematically analyzed and synthesized based on the socio-ecological model.

**Results:**

The review revealed significant deviations between traditional Tibetan feeding practices and scientific guidelines: low rates of exclusive breastfeeding, prevalent early introduction of complementary foods (median age ~1 month), primarily Tsampa, and high dietary monotony. These feeding patterns were reported to be associated with a high burden of stunting and anemia, with anemia rates exceeding 60% in some areas, and early introduction of traditional infant feeding items such as Tsampa was also described in some reports as a possible factor related to neonatal necrotizing enterocolitis (NEC). Socio-ecological analysis identified potential multi-level factors including individual knowledge gaps, community cultural norms and inadequate health services, and macro-level high-altitude environment and poverty.

**Conclusion:**

This study proposes a novel, culturally integrated intervention conceptual framework that systematically elucidates the multi-level determinants of malnutrition and provides a conceptual basis for multi-sectoral collaboration. The framework aims to reconcile traditional practices with nutritional science, highlighting the necessity of systemic, multi-level interventions, and may offer a context-sensitive conceptual reference for thinking about culturally embedded health inequities in other marginalized communities, although its applicability beyond Tibetan populations requires empirical validation in other cultural and geographic settings.

## 1 Introduction

The first 1,000 days of life, from pregnancy to a child’s second birthday, constitute a unique biological window during which nutritional provision profoundly “programs” an individual’s physiological functions, cognitive development, and long-term health. Consequently, nutritional status during this critical period directly determines an individual’s developmental potential and ultimately translates into the core engine for a nation’s human capital and sustainable development (1–3). However, the global burden of malnutrition is not evenly distributed; it is deeply rooted in complex socioeconomic and cultural contexts. From the perspective of the core public health concept of the Social Determinants of Health (SDH), an individual’s health status is determined not only by biological characteristics or personal choices but is more significantly shaped by the environments in which they live, grow, work, and age. These environments are formed by a combination of structural factors (such as income, education, gender, ethnicity, and socioeconomic policies) and intermediary factors (such as housing, food security, social support networks, and cultural norms). The SDH framework emphasizes that health inequalities stem from the uneven distribution of social resources and must be addressed through intersectoral collaboration and equitable policy interventions to promote better nutritional status and overall health equity (4). Within this framework, “Health Equity” is the pursuit of eliminating health disparities caused by social status, ethnicity, or other disadvantaged circumstances, ensuring that everyone has a fair and just opportunity to attain their full health potential (5). Therefore, the health status of vulnerable groups, such as ethnic minorities, and particularly the nutritional status of their infants and young children, not only reflects their own well-being but also serves as a critical indicator for measuring the inclusivity and effectiveness of a public health system. Health issues like malnutrition often stem from deep-seated socioeconomic inequalities and urgently require targeted policy interventions to be addressed.

Malnutrition during infancy and early childhood leads to a range of severe and potentially irreversible long-term consequences. Stunting, characterized by impaired linear growth, is not only a physical marker but is also closely associated with impaired cognitive function, reduced learning capacity, delayed school enrollment, and lower labor productivity in adulthood (2). Furthermore, nutritional deficiencies in early life, through the mechanism of “metabolic reprogramming,” significantly increase the risk of chronic non-communicable diseases such as obesity, diabetes, and cardiovascular disease in later life, creating a “life-cycle trap” of poor health (3). In China, the nutritional status of children has improved significantly in recent years, with the national stunting rate decreasing from 7.4% in 2012 to 4.7% in 2019 (6). However, in certain rural and ethnic minority regions, malnutrition remains a severe challenge. For example, research indicates that the prevalence rates of stunting and anemia among Tibetan infants and young children in areas like Qinghai are far higher than the national average; the anemia rate for children aged 6–23 months in Qinghai is as high as 59.1% (7). These health challenges not only threaten children’s immediate development and long-term well-being but also constitute a significant public health barrier to improving the region’s human capital and achieving sustainable development by impairing cognitive development and future labor productivity.

Regarding the unique traditional feeding practices for Tibetan infants and young children, existing research has described their specific patterns. Studies reveal a widespread practice in the region: the premature introduction of complementary foods, particularly simple traditional staple-based foods or preparations such as tsampa/zanba, alongside the insufficient intake of protein- and micronutrient-rich animal-source foods, such as eggs and meat. In the reviewed literature, these early complementary foods were generally described by food type rather than detailed recipe composition; available evidence suggests that they were often simple, cereal-based preparations rather than diverse mixed dishes containing multiple food groups. In this manuscript, “traditional staple-based complementary foods” refers specifically to culturally customary foods or preparations used as complementary foods during infancy, especially staple-based items such as tsampa/zanba paste, porridge, pag, or tsamtuk. It does not refer to all foods provided during the complementary feeding period. Traditional liquid or dairy feeds, such as yak milk and butter tea/bocha, are discussed separately because they may function more as liquid feeds or breast-milk/formula substitutes than as staple complementary foods. These inadequate dietary patterns are closely associated with adverse health outcomes, including infant anemia (7, 8).

Although these studies have provided valuable baseline data for understanding the current situation of the problem, they are mostly descriptive cross-sectional surveys and generally lack a macro-level public health blueprint that systematically integrates “problem analysis” with “solutions.” Specifically, few studies have employed established theoretical frameworks to systematically analyze the multi-level factors influencing these feeding behaviors and, based on such analysis, to construct comprehensive, culturally adapted intervention strategies. In light of this, using the socio-ecological model as its analytical framework, this study aims to systematically synthesize the current status and health impacts of traditional Tibetan infant feeding practices through an integrative review. A key objective is to construct a visual, culturally adapted conceptual framework for intervention, intended to provide public health policymakers and frontline practitioners in the region with a preliminary, evidence-informed reference for future intervention planning and hypothesis generation. Additionally, by using the Tibetan region as a case study, we aim to develop a context-specific conceptual framework that may generate transferable insights for addressing culturally entrenched health inequity issues in other marginalized communities, while recognizing that such transferability must be examined in future studies across different cultural and geographic settings.

Although this study is grounded in the socio-ecological model and informed by the social determinants of health perspective, its conceptual contribution does not lie in replacing these established frameworks. Rather, the proposed framework extends them by specifying how multi-level determinants can be translated into culturally acceptable and locally actionable intervention strategies. In this manuscript, “Cultural Translation” refers to the process of reconciling biomedical nutrition recommendations with culturally meaningful feeding practices, so that traditional practices are not simply rejected, but are critically modified to reduce nutritional risks while preserving their social acceptability. This concept therefore provides an operational bridge between determinant analysis and intervention design.

## 2 Methods

### 2.1 Analytical framework

This study is theoretically grounded in the classic Socio-Ecological Model (SEM). Developed by Urie Bronfenbrenner in 1979, the model’s core premise is that individual behavior is not a result of isolated personal choices but is deeply embedded within, and complexly influenced by, multiple nested environmental systems ranging from the micro to the macro level (9). In this study, the socio-ecological model was used primarily as an analytical scaffold to identify determinants operating at different levels. “Cultural Translation” was then used as an intervention-oriented concept to guide how these determinants could be addressed in culturally acceptable ways. Thus, while SEM helps explain where feeding-related risks are located, Cultural Translation helps specify how public health recommendations can be reformulated and embedded within local food practices, family decision-making, community norms, and health-service delivery. Using this analytical framework, this study systematically examines the multi-level factors influencing traditional feeding behaviors among Tibetan infants and young children. These factors span from the individual and interpersonal levels (e.g., a mother’s knowledge and beliefs, influence from family members), to the community and organizational levels (e.g., local cultural norms, accessibility of health services), and up to the macro-policy and environmental levels (e.g., socioeconomic conditions, public health policies). Therefore, the framework not only guides our analysis of the current state of feeding patterns but also provides a critical lens through which to reveal how factors at different levels intertwine to collectively shape current feeding practices and their potential implications for child nutritional health.

### 2.2 Literature search

#### 2.2.1 Search strategy

A search was conducted using a combination of subject headings and free-text words in the following databases: PubMed, Web of Science, Scopus, Embase, CINAHL, China National Knowledge Infrastructure (CNKI), and the WanFang Data Knowledge Service Platform. In addition, Google Scholar was searched to identify potentially relevant peer-reviewed publications and to support backward citation searching. The time frame for the literature search extended from the inception of each database to August 31, 2025. Search terms were organized into five main modules based on the research questions, and both English and Chinese keywords were used to ensure the comprehensiveness of the search. The specific keywords are as follows:

Population: Tibetan OR Tibetans OR “Tibetan ethnic” OR “Tibetan Plateau.”

Core Topic: “infant feeding” OR breastfeeding OR breastfeed OR “exclusive breastfeeding” OR “complementary food” OR “complementary feeding” OR “infant and young child feeding” OR IYCF OR weaning.

Specific Foods: tsampa OR zanba OR “yak milk” OR “butter tea” OR bocha.

Health Outcomes: nutrition OR nutrit OR malnutrit OR stunting OR malnutrition OR “growth and development” OR “child growth” OR anemia OR anaemia OR rickets. Although anemia and stunting were the primary nutrition-related outcomes emphasized in this review, “rickets” was retained as a secondary nutrition-related growth and micronutrient-deficiency outcome because vitamin D/calcium-related disorders may also reflect nutritional vulnerability among infants and young children in high-altitude Tibetan-inhabited areas. Including this term helped improve the sensitivity of the search and reduced the risk of missing relevant studies on broader nutrition-related health outcomes.

Public Health & Intervention Perspective: “public health” OR “community intervention” OR “health policy” OR “health disparity” OR “health equity” OR “health education” OR “cultural competence” OR “cultural adaptation” OR “cultural competency” OR “Social Determinants of Health”.

The construction of the search strategy follows the principle of “modular combination.” First, within each keyword module, the Boolean logical operator “OR” is used to connect all synonymous or related search terms (including subject headings and free-text words) to build five comprehensive concept sets. Subsequently, “AND” is used to combine the “Study Population” module with all other thematic modules (which are connected to each other by “OR”). Finally, infant age limiters are added using “AND,” and literature on irrelevant age groups (such as adults or adolescents) is excluded using “NOT.” The final search logic is: Study Population AND (Core Theme OR Specific Foods OR Health Outcomes OR Public Health & Intervention Perspectives) AND (infant OR child OR baby) NOT (adult OR adolescent). This strategy ensures that every retrieved article is relevant to the study population, pertains to at least one of the core research topics, and is limited to the infant and young child age group. The complete search strategy, as applied to PubMed, is shown in Table 1.

**Table 1:** PubMed database search strategy.

Step	Search query	Description
#1	(“Tibetan”[Title/Abstract] OR “Tibetans”[Title/Abstract] OR “Tibetan ethnic”[Title/Abstract] OR “Tibet”[Title/Abstract] OR “Tibetan Plateau”[Title/Abstract] OR “Qinghai”[Title/Abstract])	Population: Targets the Tibetan ethnic group or major Tibetan-inhabited regions.
#2	(“Infant Nutritional Physiological Phenomena” [Mesh] OR “Breast Feeding” [Mesh] OR “Weaning”[Mesh] OR “infant feeding” [Title/Abstract] OR “child feeding”[Title/Abstract] OR “complementary feeding” [Title/Abstract] OR “complementary food”[Title/Abstract] OR “breastfeed” [Title/Abstract] OR “breastfeeding”[Title/Abstract] OR “exclusive breastfeeding” [Title/Abstract] OR “weaning” [Title/Abstract])	Core Topic: Core concepts related to infant and young child feeding.
#3	(“tsampa”[Title/Abstract] OR “zanba”[Title/Abstract] OR “yak milk”[Title/Abstract] OR “butter tea”[Title/Abstract] OR “bocha”[Title/Abstract])	Specific Foods: Includes keywords for foods characteristic of the Tibetan diet.
#4	(“Malnutrition”[Mesh] OR “Infant Nutrition Disorders”[Mesh] OR “Anemia”[Mesh] OR “Growth Disorders”[Mesh] OR “Rickets”[Mesh] OR nutrition[Title/Abstract] OR nutrit[Title/Abstract] OR stunting[Title/Abstract] OR malnutrition[Title/Abstract] OR malnutrit[Title/Abstract] OR anemia[Title/Abstract] OR anaemia[Title/Abstract] OR rickets[Title/Abstract] OR “growth and development”[Title/Abstract] OR “child growth”[Title/Abstract])**	Health Outcomes: Health issues related to feeding practices and broader nutrition-related growth or micronutrient-deficiency outcomes. Rickets was included as a secondary outcome-related term to capture vitamin D/calcium-related nutritional disorders that may be relevant to infant and young child nutrition in high-altitude Tibetan-inhabited areas.
#5	(“Health Education”[Mesh] OR “Community Health Services”[Mesh] OR “Public Health”[Title/Abstract] OR “Community Intervention”[Title/Abstract] OR “Health Policy”[Title/Abstract] OR “Health Disparities”[Title/Abstract] OR “Health Education”[Title/Abstract] OR “Cultural Competency”[Title/Abstract] OR “cultural competence”[Title/Abstract] OR “cultural competency”[Title/Abstract] OR “cultural adaptation”[Title/Abstract] OR “health equity”[Title/Abstract])	Public Health & Intervention Angle: Covers concepts related to interventions and policies.
#6	#2 OR #3 OR #4 OR #5	Combination of All Topics: All concepts related to the research question (feeding, foods, outcomes, interventions) are combined using “OR.”
#7	#1 AND #6 AND (“infant”[Title/Abstract] OR “child”[Title/Abstract] OR “baby”[Title/Abstract] OR “young child”[Title/Abstract])	Final Combination: The population set (#1) is combined with the combined topics set (#6) using “AND.” Then, age limiters are added using “AND” and “NOT” to ensure the results are precisely focused on the target population and topic.

#### 2.2.2 Literature inclusion and exclusion criteria

To ensure that the literature ultimately included in the analysis is highly relevant to the research question, we have established the following strict inclusion and exclusion criteria. The literature screening process was conducted independently by two researchers, and any disagreements were resolved through discussion or by consulting a third researcher.

Inclusion criteria:

(1) Study Type: Published original empirical research, including quantitative studies (e.g., cross-sectional, cohort studies), qualitative studies (e.g., phenomenology, ethnography), and mixed-methods studies.
(2) Population: Study participants are infants and young children (explicitly defined as from birth to 24 months of age, the key window of the first 1,000 days of life) and/or their primary caregivers (e.g., parents, grandparents) living in Tibetan-inhabited areas of China (such as Tibet, Qinghai, etc.). In this review, “primary caregivers” were defined as individuals who were mainly responsible for the infant’s or young child’s daily feeding and care, rather than being limited to biological mothers or parents. Therefore, caregivers could include mothers, fathers, grandparents, other relatives, neighbors, or paid domestic helpers, provided that they were the main persons responsible for daily feeding and care.
(3) Phenomenon of Interest: The core content of the literature must focus on one or more of the following themes: feeding practices of Tibetan infants and young children, including breastfeeding, complementary feeding, the use of traditional staple-based complementary foods such as tsampa/zanba, and the use of traditional liquid or dairy feeds such as yak milk and butter tea/bocha; multi-level factors influencing feeding behaviors; or health outcomes directly related to feeding methods or broader nutrition-related growth and micronutrient deficiencies (e.g., stunted growth, anemia, and rickets).
(4) Language & Publication Status: Papers published in Chinese or English in peer-reviewed journals.

Exclusion criteria:

(1) Non-original research: Including all types of reviews, commentaries, editorials, conference abstracts, theses/dissertations, and book chapters.
(2) Mismatched Population: The study population is not Tibetan, or valid data specifically on the Tibetan population cannot be extracted separately from the article. Studies involving non-maternal caregivers were not excluded if those caregivers were the primary persons responsible for the child’s daily feeding and care.
(3) Irrelevant Content: Studies not directly related to infant and young child feeding, nutrition, or associated health outcomes.
(4) Duplicate Publications: For articles with duplicate data, only the one with the most comprehensive information and complete publication was retained.

### 2.3 Literature quality appraisal

To systematically assess the rigor and reliability of the included studies, this research utilized corresponding standardized critical appraisal tools for quality evaluation based on the different study designs. Specifically, we employed checklists from the UK’s Critical Appraisal Skills Programme (CASP) series, including the Qualitative Studies Checklist, Cohort Study Checklist, Case–Control Study Checklist, and Cross-sectional Study Checklist (10). For case series studies, the Critical Appraisal Checklist for Case Series from the Joanna Briggs Institute (JBI) in Australia was used (11, 12). The appraisal was conducted independently by two researchers. Before beginning the evaluation, both researchers first conducted a pilot appraisal on 2–3 articles to ensure a consistent understanding of the criteria. During the process, any disagreements were resolved through discussion and negotiation; if a consensus could not be reached, a third researcher was brought in to adjudicate. This study performed a quality appraisal on all included literature, but no articles were excluded solely due to low quality. Instead, the appraisal results were used in the results synthesis and discussion sections to assess the overall strength of the evidence, explain potential heterogeneity among findings, and illustrate the limitations of the existing evidence. The detailed quality appraisal results for all articles are shown in Tables 2–6.

**Table 2:** Critical appraisal results for cross-sectional studies.

First author	1. Did the study address a clearly focused issue?	2. Did the authors use an appropriate method to answer their question?	3. Were the subjects recruited in an acceptable way?	4. Were the measures accurately measured to reduce bias?	5. Were the data collected in a way that addressed the research issue?	6. Did the study have enough participants to minimize the play of chance?	7. How are the results presented and what is the main result?	8. Was the data analysis sufficiently rigorous?	9. Is there a clear statement of findings?	10. Can the results be applied to the local Population?	11. How valuable is the research?
Chen et al. and (14) Chang (15)	Yes	Yes	Cannot Tell	Yes	Yes	Cannot Tell	Yes	Cannot Tell	Yes	Yes	Yes
Chang (15)	Yes	Yes	Cannot Tell	Yes	Yes	Yes	Yes	Yes	Yes	Cannot Tell	Cannot Tell
Cidan et al. (16)	Yes	Yes	No	Yes	Yes	Yes	Yes	Yes	Yes	No	Yes
He et al. (17)	Yes	Yes	Cannot Tell	Yes	Yes	Yes	Yes	Yes	Yes	Cannot Tell	Yes
Huang et al. (18)	Yes	Yes	Cannot Tell	Yes	Yes	Yes	Yes	Yes	Yes	Cannot Tell	Yes
Li ^a^ et al. (19)	Yes	Yes	Yes	Yes	Yes	Yes	Yes	Yes	Yes	Yes	Yes
Li ^b^ et al. (20)	Yes	Yes	Yes	Yes	Yes	Yes	Yes	Yes	Yes	Yes	Yes
Lian et al. (21)	Yes	Yes	Yes	Yes	Yes	Yes	Yes	Yes	Yes	Yes	Yes
Liu et al. (22)	Yes	Yes	Yes	Yes	Yes	Yes	Yes	Yes	Yes	Yes	Yes
Nima et al. (23)	Yes	Yes	No	Yes	Yes	Yes	Yes	Yes	Yes	No	Yes
Nima et al. (24)	Yes	Yes	Yes	Yes	Yes	Yes	Yes	Yes	Yes	Yes	Yes
Wang et al. (25)	Yes	Yes	Yes	Yes	Yes	Yes	Yes	Yes	Yes	Yes	Yes
Wang et al. (26)	Yes	Yes	Yes	Yes	Yes	Yes	Yes	Yes	Yes	Cannot Tell	Yes
Wang et al. (27)	Yes	Yes	Yes	Yes	Yes	Yes	Yes	Yes	Yes	Cannot Tell	Yes
Wang et al. (28)	Yes	Yes	Yes	Yes	Yes	Cannot Tell	Yes	Yes	Yes	Cannot Tell	Yes
Xie et al. (29)	Yes	Yes	Yes	Yes	Yes	Yes	Yes	Yes	Yes	Cannot Tell	Yes
Xing et al. (30)	Yes	Yes	Yes	Yes	Yes	Yes	Yes	Yes	Yes	Cannot Tell	Yes
Xu et al. (31)	Yes	Yes	Yes	Yes	Yes	Yes	Yes	Yes	Yes	Cannot Tell	Yes
Yang et al. (32)	Yes	Yes	Yes	Yes	Yes	Yes	Yes	Yes	Yes	Cannot Tell	Yes
Yang et al. (33)	Yes	Yes	Yes	Yes	Yes	Yes	Yes	Yes	Yes	Cannot Tell	Yes
Yue et al. (34)	Yes	Yes	Yes	Yes	Yes	Yes	Yes	Yes	Yes	Cannot Tell	Yes
Zhaxi et al. (35)	Yes	Yes	Yes	Yes	Yes	Yes	Yes	Yes	Yes	Cannot Tell	Yes
Zhang et al. (36)	Yes	Yes	Yes	Yes	Yes	Yes	Yes	Yes	Yes	Cannot Tell	Yes
Dang et al. (8)	Yes	Yes	Yes	Yes	Yes	Yes	Yes	Yes	Yes	Cannot Tell	Yes
Huang ^a^ et al. (7)	Yes	Yes	Yes	Yes	Yes	Yes	Yes	Yes	Yes	Cannot Tell	Yes
Huang ^b^ et al. (37)	Yes	Yes	Yes	Yes	Yes	Yes	Yes	Yes	Yes	Cannot Tell	Yes
Wang ^a^ et al. (38)	Yes	Yes	Yes	Yes	Yes	Yes	Yes	Yes	Yes	Cannot Tell	Yes
Wang ^b^ et al. (39)	Yes	Yes	Yes	Yes	Yes	Yes	Yes	Yes	Yes	Cannot Tell	Yes
Ye et al. (40)	Yes	Yes	Yes	Yes	Yes	Yes	Yes	Yes	Yes	Cannot Tell	Yes
Li et al. (6)	Yes	Yes	Yes	Yes	Yes	Cannot Tell	Yes	Yes	Yes	No	Yes

**Table 3:** JBI (Joanna Briggs Institute) critical appraisal results for case series.

First author	1. Were there clear criteria for inclusion in the case series?	2. Was the condition measured in a standard, reliable way for all participants included in the case series?	3. Were valid methods used for identification of the condition for all participants included in the case series?	4. Did the case series have consecutive inclusion of participants?	5. Did the case series have complete inclusion of participants?	6. Was there clear reporting of the demographics of the participants in the study?	7. Was there clear reporting of clinical information of the participants?	8. Were the outcomes or follow up results of cases clearly reported?	9. Was there clear reporting of the presenting site(s)/clinic(s) demographic information?	10. Was statistical analysis appropriate?
Dawa et al. (41)	Yes	Yes	Yes	Unclear	Unclear	Yes	Yes	Yes	Yes	Yes
Wu et al. (42)	Yes	Yes	Yes	Unclear	Unclear	Yes	Yes	Not applicable	Yes	Yes

**Table 4:** Results of critical appraisal for qualitative studies.

First author	1. Was there a clear statement of the aims of the research?	2. Is a qualitative methodology appropriate?	3. Was the research design appropriate to address the aims of the research?	4. Was the recruitment strategy appropriate to the aims of the research?	5. Was the data collected in a way that addressed the research issue?	6. Has the relationship between researcher and participants been Adequately considered?	7. Have ethical issues been taken into consideration?	8. Was the data analysis sufficiently rigorous?	9. Is there a clear statement of findings?	10. How valuable is the research?
Guosheng et al. (43)	Yes	Yes	Yes	Yes	Yes	No	No	No	Yes	Yes

**Table 5:** Results of critical appraisal for cohort studies.

First author	1. Did the study address a clearly Focused issue?	2. Was the cohort recruited In an acceptable way?	3. Was the exposure accurately Measured to minimize bias?	4. Was the outcome accurately Measured to minimize bias?	5. (a) Have the authors identified All important confounding factors?	5. (b) Have they taken account of the confounding factors in the design and/or analysis?	6. (a) Was the follow up of subjects complete enough?	6. (b) Was the follow up of subjects long enough?	7. What are the results of this study?	8. How precise are the results?	9. Do you believe the results?	10. Can the results be applied to the local population?	11. Do the results of this study fit with other available evidence?	12. What are the implications of this study for practice?
Liu et al. (44)	Yes	No	Yes	Yes	No	No	Yes	Yes	Yes	Yes	No	No	Yes	Yes

**Table 6:** Results of critical appraisal for case–control studies.

First author	1. Did the study address a clearly focused issue?	2. Did the authors use an appropriate method to answer their question?	3. Were the cases recruited in an acceptable way?	4. Were the controls selected in an acceptable way?	5. Was the exposure accurately Measured to minimize bias?	6a. Aside from the experimental intervention, were the groups treated equally?	6b. Have the authors taken account of the potential confounding factors in the design and/or in their analysis?	7. How large was the treatment effect?	8. How precise was the estimate of the treatment effect?	9. Do you believe the results?	10. Can the results be applied to the local population?	11. Do the results of this study fit with other available evidence?
Huang ^c^ et al. (45)	Yes	No	Yes	Yes	Yes	Yes	Yes	Yes	Yes	Yes	Cannot Tell	Yes

### 2.4 Literature screening, data extraction, and analytical synthesis

The literature screening process was conducted in strict accordance with the PRISMA (Preferred Reporting Items for Systematic Reviews and Meta-Analyses) guidelines (13). First, the search results from all databases were imported into reference management software (e.g., Zotero), where duplicates were removed using a combination of automatic and manual checks. Following this, two researchers independently screened the articles in two stages:

(1) Stage One (Title and Abstract Screening): Based on the titles and abstracts, studies that were clearly irrelevant were preliminarily excluded.
(2) Stage Two (Full-Text Screening): The full texts of the articles remaining after the initial screening were carefully read to make a final determination of eligibility based on the inclusion and exclusion criteria outlined in section “inclusion and exclusion criteria”.

Throughout the screening process, any disagreements between the researchers were resolved through discussion or consultation with a third researcher. The final literature screening process and results are presented in a PRISMA flow diagram.

Data extraction was carried out using a pre-designed, standardized data extraction form. Similarly, two researchers independently extracted the information and cross-checked their results to ensure the accuracy and completeness of the data. The key information extracted included:

(1) Basic Information: First author, year of publication, journal.
(2) Study Characteristics: Study design, geographical location where the study was conducted, sample size, and participant characteristics, including caregiver type when reported.
(3) Key Research Findings: ① Feeding Practices: Specific quantitative data and qualitative descriptions regarding exclusive breastfeeding, complementary feeding (timing, types), and the use of traditional staple-based complementary foods and liquid/dairy feeds (e.g., tsampa/zanba, porridge, yak milk, and butter tea/bocha). ② Health Outcomes: Prevalence rates or evidence of association for nutrition-related problems such as stunting, anemia, and rickets. ③ Influencing Factors: Factors reported in the literature that influence feeding behaviors at the individual, family, community, and macro-policy levels. ④ Intervention Strategies: Any existing or proposed intervention measures mentioned in the literature.

**Figure 1:**
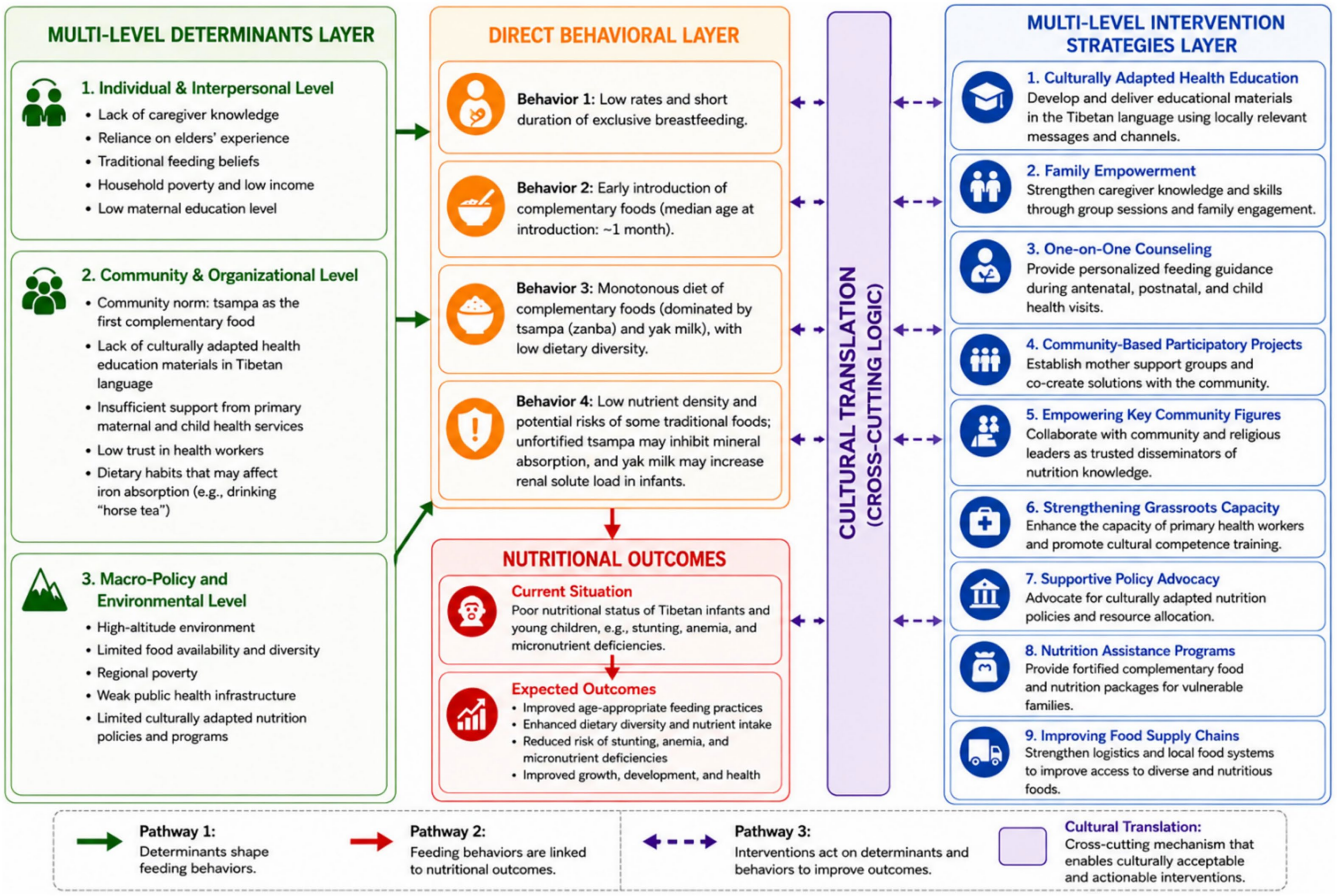
A culturally adapted conceptual framework for improving nutrition among Tibetan infants and young children based on the socio-ecological model and the concept of Cultural Translation. The framework illustrates how multi-level determinants at the individual/interpersonal, community/organizational, and macro-policy/environmental levels shape direct feeding behaviors and subsequently influence nutritional outcomes. Cultural Translation serves as a cross-cutting mechanism that transforms biomedical nutrition recommendations into culturally acceptable and locally actionable multi-level intervention strategies, thereby promoting age-appropriate feeding, dietary diversity, and improved nutritional status. Green arrows indicate pathways from determinants to feeding behaviors, red arrows indicate the link between feeding behaviors and nutritional outcomes, and purple dashed arrows indicate how intervention strategies act through Cultural Translation to address determinants and behaviors and improve outcomes.

After data extraction, an analytical synthesis was conducted to generate the conceptual framework. First, all extracted findings were organized into an evidence matrix according to four domains: feeding practices, health outcomes, influencing factors, and intervention implications. Second, feeding-related findings were inductively grouped into recurring themes, including breastfeeding patterns, timing of complementary feeding, use of traditional staple-based complementary foods such as tsampa/zanba and traditional liquid/dairy feeds such as yak milk, dietary diversity, and nutrition-related health outcomes. Third, the reported influencing factors were deductively mapped onto the socio-ecological model, including the individual/interpersonal, community/organizational, and macro-policy/environmental levels. Fourth, relationships among themes were established by tracing how specific determinants were linked in the reviewed studies to feeding behaviors and subsequent health outcomes. Finally, intervention implications were derived by matching each major determinant with a culturally appropriate and feasible intervention target. Through repeated comparison and discussion among the research team, these evidence-based themes, categories, and relationships were integrated into the final conceptual framework shown in Figure 1.

To enhance the conceptual credibility of the framework, the preliminary framework was reviewed and discussed within the research team after its initial development. The team examined whether the proposed themes, categories, and directional relationships were consistently supported by the extracted evidence and whether the framework was coherent with the socio-ecological model, the social determinants of health perspective, and the concept of Cultural Translation. This process functioned as an internal consistency check and theoretical triangulation rather than formal external validation. No formal expert consultation, stakeholder review, or community-based validation was conducted at this stage.

## 3 Results

### 3.1 Literature search results

(1) The initial database search yielded a total of 1,737 articles, comprising 1,284 from Chinese databases and 453 from English databases.
(2) Using Zotero reference management software to screen for duplicates, 40 duplicate articles were removed (5 in Chinese, 35 in English). After deduplication, a total of 1,697 articles entered the screening stage.
(3) By reading titles and abstracts, 1,574 articles that were clearly inconsistent with the inclusion criteria were excluded. The main reasons for exclusion were: ① Ineligible document type (e.g., various reviews, commentaries, editorials, conference papers, theses, patents, and book chapters), *n* = 243; ② Irrelevant research content (not directly related to infant and young child feeding, nutrition, or health outcomes), *n* = 1,322; ③ Mismatched study population (not a Tibetan population or relevant data could not be extracted), *n* = 9.
(4) A total of 123 articles (49 in Chinese, 74 in English) proceeded to the full-text reading stage for eligibility assessment. After careful reading of the full texts, 89 articles were excluded for the following specific reasons: full text unavailable (n = 2), unable to extract valid data on the Tibetan population (*n* = 5), research content not aligned with the core focus of this review (*n* = 24), and duplicate data publication (retaining only the most comprehensive article) (*n* = 58). After this stage, 34 articles remained.
(5) Additionally, one more article was included through backward citation searching of reference lists.

**Figure 2:**
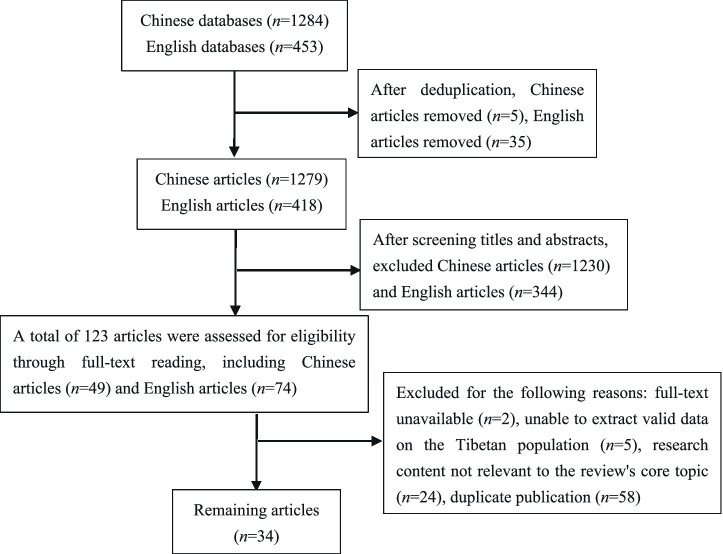
Flowchart of literature search and screening process.

Therefore, after the above screening process, a final total of 35 articles that met the criteria for this study were included, consisting of 27 Chinese articles and 8 English articles. The detailed literature selection process is shown in Figure 2, the results of the quality appraisal are presented in Tables 2–6, and the basic characteristics of the included literature are shown in Table 7.

**Table 7:** Basic information of the included literature.

First author	Publication year	Journal	Study design	Geographical location	Sample size (n)	Characteristics of participants	Key findings
Chang et al. (15)	2013	Chinese Journal of School Health	Cross-sectional study	High-altitude area (Qinghai)	1,099	0–6 year old Tibetan and Hui children (567 Tibetan, 532 Hui)	·Health Outcomes: The overall anemia prevalence in Tibetan children was 27.86%, significantly higher than the 19.55% in Hui children.
Chen et al. (14)	2024	Journal of High Altitude Medicine	Cross-sectional study	Chengduo County, Qinghai Province	151	Infants aged 0–8 months and their families	·Feeding Practices: The exclusive breastfeeding rate was low (22.52%), and the introduction of complementary foods was generally too early, with 55.63% of infants having complementary foods added between 3 and 6 months of age. Cow’s milk was often used to supplement insufficient breast milk instead of formula. ·Influencing Factors: There was a lack of feeding knowledge within families, with 64.90% of parents being illiterate. Knowledge primarily came from the experience of elders (39.74%). ·Intervention Strategies: It is recommended to create Tibetan-language educational videos on feeding knowledge and to conduct continuous health education through WeChat groups.
Cidan et al. (16)	2021	Chinese Journal of Child Health Care	Cross-sectional study	Lhasa City, Tibet	1,995	Children aged 2–6 in nursery/kindergarten (88.52% Tibetan)	·Health Outcomes: The overall anemia prevalence was as high as 68.57%, with a stunting rate of 8.37% and an underweight rate of 22.06%. The anemia rate in Tibetan children (71.07%) was significantly higher than in non-Tibetan children (49.34%).
Dang et al. (8)	2005	European Journal of Clinical Nutrition	Cross-sectional study	Tibet	1,655	Children under 36 months of age	·Feeding Practices: Complementary foods were introduced too early, with a median introduction time of around 1 month of age; the main foods were tsampa and porridge. By 6 months of age, less than 25% of children had been introduced to protein-rich foods such as eggs and meat. The exclusive breastfeeding rate was low, with the rate at 4 months being only 20.1%.
He et al. (17)	2022	Medical Journal of West China	Cross-sectional study	Kangbei Plateau, Sichuan (Garzê Prefecture)	634	Infants and young children aged 6–36 months	·Health Outcomes: The anemia prevalence was as high as 60.9%, of which 54.4% was iron-deficiency anemia. ·Influencing Factors: Multivariate analysis showed that age, gender, ethnicity, feeding method, and caregiver’s education level were independent influencing factors for anemia. The study found no significant association between the timing, type, or quantity of complementary food introduction and anemia.
Huang ^a^ et al. (7)	2019	BMJ Open	Cross-sectional study	Huzhu County, Qinghai Province	754 (including 47 Tibetan cases)	Children aged 6–23 months	·Health Outcomes: The anemia prevalence was as high as 59.1%. ·Feeding Practices and Influencing Factors: Risk factors for anemia were Tibetan ethnicity (OR = 3.123) and not adding meat to the diet (OR = 0.698; this is presented inversely, as adding meat is a protective factor). The primary cause was iron-deficiency anemia, which accounted for 80.9% of all anemia cases.
Huang ^b^ et al. (37)	2022	Journal of Clinical Laboratory Analysis	Cross-sectional study	Garzê Tibetan Autonomous Prefecture, Sichuan Province	1,815	0–6 year old Tibetan children	·Health Outcomes: The prevalence of Vitamin A deficiency (VAD) was 8.04%, and the combined incidence of vitamin D deficiency and insufficiency was high. ·Influencing Factors: The 0–1 year old infant group had the highest rates of vitamin A and D deficiency. Altitude was a significant influencing factor; the higher the altitude, the lower the vitamin A levels. Levels of vitamins A, D, and E were all highest in the summer.
Huang et al. (18)	2021	Chinese Journal of Child Health Care	Cross-sectional study	Garzê Prefecture, Sichuan Province	2,122	0–6 year old Tibetan children	·Health Outcomes: The rate of subclinical vitamin A deficiency in children was 8.15%, and the rate of marginal deficiency was 45.99%. ·Influencing Factors: Multivariate analysis showed that age (with the highest deficiency rate in 0–1 year old infants), altitude (the higher the altitude, the higher the deficiency rate), and season were the main influencing factors for vitamin A deficiency.
Li ^a^ et al. (19)	2021	Journal of Hygiene Research	Cross-sectional study	Rural areas of Sichuan Province (including 2 Tibetan counties)	1,087 (including 274 Tibetan cases)	Infants and young children aged 0–18 months and their mothers	·Feeding Practices: The exclusive breastfeeding rate within the first 6 months of life was 35.94%. ·Influencing Factors: Multivariate analysis showed that the mother’s ethnicity, delivery method, parity, family economic status, and whether the mother possessed scientific feeding knowledge were all significant factors influencing breastfeeding behavior. ·Intervention Strategies: It is necessary to improve mothers’ knowledge levels through targeted health education in order to improve the current state of breastfeeding.
Li ^b^ et al. (20)	2021	Journal of Sichuan University (Medical Science Edition)	Cross-sectional study	Multi-ethnic rural areas of Sichuan Province (including 2 Tibetan counties)	1,117 (including 330 Tibetan cases)	Infants and young children aged 12–24 months and their caregivers	·Feeding Practices: In Tibetan areas, 47.58% of infants and young children were introduced to complementary foods before 6 months of age. The first type of complementary food introduced was most commonly meat, vegetables, or fruit (42.12%). ·Health Outcomes: The malnutrition rate among infants and young children in Tibetan areas was 24.85%. ·Feeding Practices and Health Outcomes: Multivariate analysis showed that, compared to first introducing other types of complementary foods, first introducing iron-fortified rice cereal reduced the risk of malnutrition in infants and young children (OR = 0.54).
Lian et al. (21)	2009	Maternal and Child Health Care of China	Cross-sectional study	Aba Tibetan and Qiang Autonomous Prefecture, Sichuan Province	1,263 (including 700 Tibetan cases)	Children under 7 years of age	·Health Outcomes: The overall anemia prevalence was 23.91%. Tibetan children had the highest anemia prevalence, at 28.29%. ·Influencing Factors: The discussion section pointed out that anemia is related to the monotonous dietary structure in ethnic minority areas (e.g., a diet based mainly on flour-based foods), low intake of vegetables and fruits, and the habit of drinking “horse tea” (which can inhibit iron absorption).
Liu et al. (22)	2022	Food and Nutrition in China	Cross-sectional study	Contiguous impoverished areas (including Tibetan areas across four provinces)	44,543 (including 1,640 cases from the Tibetan areas of four provinces)	Infants and young children aged 6–23 months	·Health Outcomes: Among all the impoverished areas surveyed, the nutritional problems of infants and young children in the Tibetan areas of four provinces were the most prominent. Their anemia prevalence was as high as 66.2% and the stunting rate was 15.9%, both of which were far higher than the average levels for all surveyed areas (26.4 and 7.7%, respectively).
Li et al. (6)	2023	Public Health Nutrition	Cross-sectional study	Tibet	1,975	Tibetan children aged 12–59 months	·Feeding Practices: The study assessed “optimal feeding practices,” which included breastfeeding, complementary feeding, dietary diversity, and frequency. Compared to rural children, urban children had a higher rate of exclusive breastfeeding within 6 months (69.8% vs. 57.6%) and better dietary diversity. ·Health Outcomes: Overall, the stunting rate was 18.4% and the underweight rate was 8.2%. The stunting rate (21.1%) and underweight rate (9.2%) of rural children were significantly higher than those of urban children (4.6 and 3.0%, respectively). There was no statistically significant difference in the wasting rate between urban and rural areas. ·Influencing Factors: Altitude was a major risk factor for malnutrition. For every 1,000-meter increase in altitude, the risk of stunting and being underweight in children approximately doubled (ORs were 2.03 and 2.04, respectively). The risk increased rapidly above an altitude of 3,500 meters.
Nima et al. (23)	2022	Laboratory Medicine and Clinic	Cross-sectional study	Lhasa area, Tibet	1,196	Tibetan children aged 0–12 years	·Health Outcomes: The vitamin D nutritional status of children was generally poor, with a total of 78.4% of children being deficient (24.3% severely deficient, 26.9% deficient) or insufficient (27.2%). ·Influencing Factors: Vitamin D levels were significantly influenced by age, sex, and season, with deficiency and insufficiency being most severe in the winter.
Nima et al. (24)	2021	Modern Preventive Medicine	Cross-sectional study	Tibet	3,136	Children aged 0–5 years	·Health Outcomes: The child stunting rate was 15.37%. ·Feeding Practices and Health Outcomes: Multivariate analysis showed that, compared to children breastfed for less than 6 months, the risk of stunting was paradoxically higher in children who were breastfed for 12–24 months (OR = 1.91) and for ≥24 months (OR = 1.75). This suggests that while breastfeeding is prolonged, there may be problems with inadequate or inappropriate complementary feeding.
Wang et al. (25)	2013	Chinese Journal of Preventive Medicine	Cross-sectional study	Rural areas of Lhasa City, Tibet	540	Tibetan infants and young children aged 6–35 months	·Feeding Practices and Health Outcomes: The Infant and Child Feeding Index (ICFI) was used for a comprehensive assessment, which found that the overall feeding practices for infants and young children in this region were poor. The ICFI score was positively correlated with the Height-for-Age Z-score (HAZ), meaning that the higher the quality of feeding, the better the child’s linear growth (i.e., less severe stunting). However, the ICFI showed no significant association with weight-related indicators (WAZ, WHZ).
Wang ^a^ et al. (38)	2010	BMC Public Health	Cross-sectional study	Rural areas of Lhasa City, Tibet	386	Lactating mothers	·Influencing Factors: Dietary surveys of Tibetan mothers show that their dietary structure is relatively monotonous, consisting mainly of staple foods and traditional Tibetan food. Their vitamin intake is generally lower than the national average, which may affect the nutritional status of their infants through breast milk. Mothers with a higher level of education have significantly higher intakes of vitamin A and vitamin C.
Wang ^b^ et al. (39)	2015	International Journal of Environmental Research and Public Health	Cross-sectional study	Rural areas of Lhasa City, Tibet	386	Infants and young children under 24 months of age	·Feeding Practices and Health Outcomes: The study created a feeding practice index to evaluate feeding behaviors. The results showed that good feeding practices were associated with a reduced risk of acute upper respiratory tract infections in infants and young children (OR = 0.43). The study suggests that appropriate infant and young child feeding practices may play a protective role in the health of Tibetan infants.
Wang et al. (26)	2023	Gansu Medical Journal	Cross-sectional study	High-altitude area / Plateau region	1,726 (including 236 Tibetan cases)	Children aged 0–6 years	·Health Outcomes: Tibetan children had the highest anemia prevalence at 64.41%, which was significantly higher than that of Han children (44.07%) and Hui children (25.20%). ·Influencing Factors: For Tibetan children, risk factors for anemia were being under 1 year of age, having a monthly per capita family income of less than 2000 yuan, and a low frequency of vegetable and fruit intake (fewer than 4 days per week).
Wang et al. (27)	2022	Journal of Chinese Academy of Medical Sciences	Cross-sectional study	Multi-ethnic rural areas of Sichuan Province (including 2 Tibetan counties)	1,092 (including 291 Tibetan cases)	Children aged 18–36 months	·Feeding Practices: The Dietary Diversity Score (DDS) for Tibetan children was 4.9, significantly lower than that of Han children (5.8). ·Health Outcomes: The stunting rate for Tibetan children was 7.2%. ·Feeding Practices and Health Outcomes: Multivariate analysis showed that the Dietary Diversity Score (DDS) was significantly positively correlated with the Height-for-Age Z-score (HAZ), meaning that the more diverse the diet, the better the child’s linear growth.
Wang et al. (28)	2020	China Modern Doctor	Cross-sectional study	Nyingchi Prefecture, Tibet	140	0–3 year old Tibetan children	·Health Outcomes: The child underweight rate was 7.9%, and the stunting rate was 11.4%. ·Feeding Practices and Health Outcomes: Multivariate analysis showed that being fed tsampa was a common risk factor for both being underweight and for stunting in children.
Xie et al. (29)	2003	Chinese Journal of Child Health Care	Cross-sectional study	Rural areas of impoverished counties in Qinghai Province	1,914 (including 542 Tibetan cases)	Children under 3 years of age	·Health Outcomes: Nutritional problems among children in this region are severe. The stunting rate for Tibetan children was 26.2%, and the underweight rate was 15.4%. The study found that the prevalence of malnutrition in children increased with age, reaching a peak after 12 months.
Xing et al. (30)	2010	Chinese Journal of Public Health	Cross-sectional study	Rural areas of Lhasa City, Tibet	386	Mothers of Tibetan infants and young children aged 0–24 months	·Feeding Practices: Complementary feeding was introduced too early and was inappropriate. Over 80% of infants were fed tsampa paste within 4 months of age, but the introduction of foods like eggs, meat, vegetables, and fruits was delayed. ·Influencing Factors: Mothers generally lacked feeding knowledge; for example, only 21.8% of mothers knew that complementary foods should be introduced between 4–6 months of age.
Xu et al. (31)	2015	Chinese Journal of Child Health Care	Cross-sectional study	Yushu Tibetan Autonomous Prefecture, Qinghai Province	1,897(malnutrition) / 897 (anemia)	Children under 3 years of age	·Health Outcomes: Data from 2012 shows that the malnutrition rate for children in this region was 17.0% (with a stunting rate of 12.2%), and the anemia prevalence in children aged 6–35 months was as high as 64.7%.
Ye et al. (40)	2022	Journal of Nutrition Education and Behavior	Cross-sectional study	Western rural areas of Sichuan Province (including 2 Tibetan counties)	1,358 (including 338 Tibetan cases)	Caregivers of infants and young children aged 6–24 months	·Intervention Strategies / Influencing Factors: This study investigated the compliance of the Ying Yang Bao (YYB) nutrition pack program. It highlights the importance of the health communication model, noting that communication among family members and trust in community health workers influence compliance with interventions like YYB. Multivariate analysis showed that obtaining information from family members (OR = 4.86) and township doctors (OR = 7.22), as well as understanding the health benefits of the nutrition packs (OR = 2.00), significantly improved compliance.
Yang ^a^ et al. (32)	2016	Maternal and Child Health Care of China	Cross-sectional study	Sakya County, Tibet	1,606	0–5 year old Tibetan children	·Health Outcomes: The rates of child stunting (46.0%), being underweight (31.2%), and wasting (28.5%) were all high. ·Influencing Factors: Multivariate analysis showed that not being breastfed and having ≥2 children under 5 years of age in the household were common risk factors for all three types of malnutrition. In addition, maternal illiteracy was a risk factor for stunting, and low annual household income was a risk factor for wasting.
Yang ^b^ et al. (33)	2016	Chinese Journal of Child Health Care	Cross-sectional study	Sakya County, Tibet	1,884(malnutrition) / 253 (anemia)	0–7 year old Tibetan children	·Health Outcomes: The nutritional status of children in this region was poor, with a stunting rate of 30.31% and an underweight rate of 28.87%. The anemia prevalence in children aged 6 months to 6 years was 48.22%. The 1–2 year old age group was the peak period for malnutrition.
Yue et al. (34)	2019	Chinese Journal of School Health	Cross-sectional study	High-altitude area	1,512	Children aged 0–6 years	·Health Outcomes: The child anemia prevalence was as high as 67.0%. ·Feeding Practices and Health Outcomes: Feeding method was significantly associated with anemia. Among infants aged 0–6 months, the anemia rates for mixed feeding (73.1%) and artificial feeding (75.0%) were significantly higher than for exclusive breastfeeding (48.9%). Picky eating was also a risk factor for anemia.
Zhaxi et al. (35)	2021	Plateau Science Research	Cross-sectional study	Tibet Autonomous Region	1,494	Children aged 0–5 years	·Feeding Practices: The median duration of exclusive breastfeeding for children was only 2 months, and the median duration of total breastfeeding was 12 months. ·Influencing Factors: Factors influencing the duration of exclusive breastfeeding included household registration type (longer in urban areas than in agricultural/pastoral areas) and the timing of complementary food introduction (longer duration for those who introduced foods after 6 months), among others.
Zhang et al. (36)	2016	Maternal and Child Health Care of China	Cross-sectional study	Impoverished rural areas of Qinghai Province	4,394	Children aged 6–23 months	·Health Outcomes: The anemia rate (67.8%) and stunting rate (12.0%) were very high, and were particularly severe in Guinan Tibetan Autonomous County (anemia rate 86.3%, stunting rate 17.0%). ·Feeding Practices: Feeding practices were poor; the breastfeeding rate after 1 year of age was only 20%, and nearly half of the children had a monotonous diet. ·Influencing Factors: Anemia and stunting were associated with factors such as ethnicity, caregiver’s education level, and household sanitation conditions (e.g., safe drinking water, sanitary toilets).
Huang ^c^ et al. (45)	2025	BMC Public Health	Case–control study	Garzê Prefecture (Tibetan area) vs. Luzhou City (non-Tibetan area), Sichuan Province	1,990 (995 Tibetan children, 995 non-Tibetan children)	Children aged 1–10 years	·Health Outcomes: The Vitamin A deficiency rate (47.7% vs. 21.0%) and Vitamin D deficiency rate (19.8% vs. 10.7%) in Tibetan children were both significantly higher than in non-Tibetan children. ·Influencing Factors: Altitude is a significant risk factor for Vitamin A and D deficiency. The study data indicated that different daily behavioral patterns in Tibetan girls made them more susceptible to Vitamin D deficiency than boys.
Dawa et al. (41)	2018	Chinese Journal of General Practice	Case series study	Shannan City, Tibet	48	Neonates with necrotizing enterocolitis (NEC)	·Feeding Practices and Health Outcomes: Among the 48 infants with NEC, 38 had been fed tsampa (Zanba). The study clearly indicates that the premature feeding of tsampa is a significant pathogenic factor for the occurrence of NEC in newborns in the Tibetan plateau region.
Wu et al. (42)	2016	Maternal and Child Health Care of China	Case series study	Tibet area / Tibetan region	186	Tibetan children hospitalized for bronchopneumonia with comorbid iron-deficiency anemia	·Health Outcomes: A clinical analysis of 186 Tibetan children with concurrent bronchopneumonia and iron-deficiency anemia showed that anemia often co-occurs with diseases such as respiratory tract infections, which exacerbates the severity of the illness.
Liu et al. (44)	2019	Chinese Pediatric Emergency Medicine	Cohort study	Nagqu City, Tibet	536	Infants and young children aged 1–36 months hospitalized for acute bronchopneumonia	·Health Outcomes: Among infants and young children hospitalized for pneumonia, the comorbidity rate of anemia was as high as 55.6%. ·Feeding Practices and Health Outcomes: The incidence of anemia was significantly correlated with the timing of the introduction of tsampa paste. The earlier tsampa paste was introduced, the higher the incidence of anemia (for example, the anemia rate reached 63.54% among infants who had it introduced at 1–3 months of age).
Guosheng et al. (43)	2008	Chinese Journal of Social Medicine	Qualitative study	Agricultural and pastoral areas of Tibet(7 counties)	336	Pregnant women, postpartum mothers, and mothers of children aged 0–5 years	·Influencing Factors (Feeding Beliefs): Deeply rooted traditional beliefs exist. Most mothers believe that newborns should be fed tsampa paste 1–2 days after birth, believing this will help the child “grow up strong.” ·Feeding Practices: Exclusive breastfeeding within the first 6 months is generally not achieved because of the belief that breast milk alone is not enough for the baby and that staple foods must be added. Complementary foods are introduced too early and lack variety; due to geographical and economic limitations, vegetables and fruits are rarely added.

OR, Odds Ratio.

### 3.2 Basic characteristics and quality appraisal summary of included studies

Among the 35 studies ultimately included, there were 30 cross-sectional surveys (6, 14–40), 1 case–control study (45), 2 case series (41, 42), 1 cohort study (44), and 1 qualitative study (43) (see Table 7 for details). In terms of geographical distribution, all studies were conducted in the Tibetan-inhabited areas of China, covering a wide region. They were primarily concentrated in the Tibet Autonomous Region (e.g., Lhasa, Sakya County), Qinghai Province (e.g., Chengduo County, Yushu Prefecture), and the Tibetan areas of Sichuan Province (e.g., Garzê Prefecture, Aba Prefecture). These studies provide multi-dimensional evidence for a comprehensive understanding of infant and young child feeding practices and their health impacts across different Tibetan communities. Regarding the research content, the focus of these publications varied: 14 studies explicitly analyzed the association between feeding behaviors (such as complementary food types, dietary diversity, feeding methods) and specific health outcomes (such as stunting, anemia, necrotizing enterocolitis) (6, 7, 20, 21, 24–28, 32, 34, 39, 41, 44); 8 studies focused on describing the current status of feeding practices, feeding knowledge, or their influencing factors, but did not directly analyze their association with health outcomes (8, 14, 19, 30, 35, 38, 40, 43); the remaining 13 studies mainly reported the prevalence of specific health outcomes (such as anemia, vitamin deficiencies) or explored influencing factors other than feeding methods (such as ethnicity, altitude) (15–18, 22, 23, 29, 31, 33, 36, 37, 42, 45). Collectively, these studies reveal common problems in the feeding of Tibetan infants and young children and provide rich data to support the exploration of the multi-level influencing factors behind them. In summary, the included studies reported stunting rates ranging from 7.2 to 46.0% (6, 27, 28, 32), anemia rates as high as 23.91 to 68.57% (16, 21, 34), and rates of exclusive breastfeeding within 6 months generally below 36% (14, 19). These data collectively point to a severe public health challenge.

The quality appraisal results indicated that the included studies were generally relevant to the review question, but their methodological quality was uneven. Most cross-sectional studies addressed clearly focused questions, used broadly appropriate designs, and reported clear findings; however, several studies had limitations related to unclear recruitment procedures, insufficient reporting of sample representativeness, limited control of confounding factors, and uncertain applicability to all Tibetan-inhabited areas. The two case series provided useful clinical observations but offered limited evidence for population-level inference. The single qualitative study contributed important contextual insights into cultural beliefs and feeding practices, but it’s reporting of researcher reflexivity, ethical considerations, and analytic rigor was limited. The cohort and case–control studies provided comparatively stronger analytical evidence than descriptive studies, but limitations in recruitment, confounding control, and generalizability remained. Therefore, the overall evidence base was considered useful for identifying recurring feeding patterns, contextual determinants, and plausible intervention targets, but less robust for establishing causal pathways or validating the proposed framework.

### 3.3 Thematic synthesis results

The thematic synthesis generated three sequential analytic domains: current feeding practices, nutrition-related health impacts, and socio-ecological determinants of feeding behaviors. The first two domains were primarily derived inductively from recurring findings across the included studies, while the determinant categories were organized deductively according to the socio-ecological model. These domains were then linked to explain how culturally embedded feeding practices, such as early introduction of tsampa and limited dietary diversity, may contribute to adverse nutritional outcomes through multi-level individual, family, community, health-system, and macro-environmental pathways.

In interpreting these themes, greater confidence was placed on findings that were repeatedly reported across multiple studies and supported by clearer measurement and reporting. Findings derived from single studies, case series, or studies with important methodological limitations were treated as suggestive rather than definitive and were used mainly to inform contextual interpretation and hypothesis generation.

#### 3.3.1 Current situation: core feeding practices of Tibetan infants and young children

Through a systematic integration of the 35 included studies, several core patterns of feeding practices for Tibetan infants and young children can be clearly delineated. These patterns are rooted in deep cultural traditions and a unique geographical environment, but they show significant deviation from the scientific feeding guidelines recommended by the World Health Organization (WHO) and China.

##### 3.3.1.1 Breastfeeding patterns: low rate of exclusive breastfeeding and insufficient duration

Although breastfeeding is a common practice, the implementation of exclusive breastfeeding is far from ideal. Both WHO and Chinese feeding guidelines recommend that the exclusive breastfeeding rate for infants under 6 months should exceed 50% (46–48). However, multiple studies indicate that the actual rate in this region falls far short of this target. For example, the exclusive breastfeeding rate in Chengduo County, Qinghai, was only 22.52% (14), and in rural areas of Sichuan (including Tibetan areas), it was only 35.94% (19). The median duration of total breastfeeding was approximately 12 months, but the median duration of exclusive breastfeeding was only 2 months (35). This pattern is associated with various factors, including the traditional belief among mothers that breast milk alone is insufficient and that staple foods need to be added (43), as well as a lack of knowledge about scientific feeding practices (14).

##### 3.3.1.2 Complementary feeding patterns: prevalent early introduction and lack of diversity

The “premature” introduction of complementary foods is an extremely prominent feeding issue in Tibetan areas. Substantial evidence indicates that many infants begin receiving complementary foods long before the recommended age of 6 months. A study in Tibet found that the median age for introducing complementary foods was only around 1 month (8), and over 80% of infants were fed “zanba” (roasted barley flour) paste within 4 months of age (30). Research in Chengduo County, Qinghai, also showed that 55.63% of infants had been given complementary foods between 3 and 6 months of age (14). Accompanying the early introduction is a highly monotonous diet. The first foods introduced are often not the recommended iron-fortified rice cereal but rather traditional infant feeding items such as zanba, porridge, yak milk, and butter tea. Most included studies reported these complementary foods as broad food categories rather than providing detailed ingredient-level recipes. Based on the available descriptions, early complementary foods appeared to be mainly simple staple-based foods or preparations, such as zanba paste or porridge, sometimes combined with liquids such as tea, milk, or butter tea, rather than nutritionally diverse mixed dishes containing multiple food groups. Even by the age of 6 months, less than 25% of children were given foods rich in high-quality protein, such as eggs and meat (8). This low intake of animal-source foods is particularly important because eggs and meat provide high-quality protein and highly bioavailable micronutrients, including heme iron, zinc, and vitamin B12, which are directly relevant to the prevention of stunting, anemia, and micronutrient deficiencies. This uniform dietary structure leads to dietary diversity scores that are significantly lower than those of other ethnic groups. For instance, one study reported that the dietary diversity score for Tibetan children was only 4.9, significantly lower than the 5.8 score for Han Chinese children (27).

##### 3.3.1.3 Nutritional characteristics and risks of core traditional infant feeding items

For clarity, the traditional infant feeding items identified in the reviewed studies can be broadly distinguished into staple-based complementary foods or preparations, represented by tsampa/zanba, and liquid or dairy feeds, represented by yak milk and butter tea/bocha. The former are mainly discussed as complementary foods, whereas the latter are discussed as traditional liquid feeds or potential breast-milk/formula substitutes.

Zanba/Tsampa: This is the core staple food in Tibetan dietary culture, made from roasted highland barley flour. It is typically consumed in the form of “pag” (a doughy ball) or “tsamtuk” (a porridge or soup). These preparations may involve simple combinations with water, tea, milk, or butter tea, but they remain largely cereal-dominant and do not usually represent diversified mixed complementary meals containing animal-source foods, vegetables, fruits, or other nutrient-dense ingredients. Zanba paste is the earliest and most frequently introduced complementary food for infants (30, 43), as traditional beliefs hold that zanba makes children “grow strong” (43). However, from a nutritional perspective, while zanba provides energy and dietary fiber, it has significant nutritional deficiencies. Firstly, its protein quality is low. Secondly, unfortified zanba contains extremely high levels of phytic acid (averaging 1,105 mg/100 g, far exceeding that of wheat flour and rice), which is a major anti-nutritional factor that significantly inhibits the body’s absorption of key minerals such as calcium, iron, and zinc (46). More importantly, introducing it prematurely as a primary complementary food may occupy an infant’s limited stomach capacity due to its low nutrient density, thereby potentially crowding out the intake of breast milk or other more nutrient-dense foods. Multiple studies indicate that the premature and excessive feeding of Tsampa (roasted barley flour) has been associated with low body weight, stunting, and anemia in children (28, 44). In extreme cases, the early feeding of Tsampa has also been reported as a possible contributing factor to neonatal necrotizing enterocolitis (NEC) (41).

*Yak milk and other dairy products*: In areas where infant formula is expensive or difficult to obtain, it is not uncommon for infants to be fed directly with animal milk such as yak milk (14), but this poses significant health risks. Compared to human breast milk, the nutritional composition of yak milk does not match the needs of an infant: its protein content (5.2 g/100 g vs. 1.0 g/100 g) and mineral content (e.g., calcium at 129 mg/100 g vs. 32 mg/100 g) are both excessively high (47). This high-protein, high-mineral composition significantly increases the solute load on an infant’s immature kidneys and can even damage the nephrons through hyperfiltration (48). Research has confirmed that early intake of high-protein formula not only leads to an increase in kidney size in infants but is also associated with higher blood pressure 11 years later (48). Therefore, overall, unmodified mammalian milk (including yak milk) is unsuitable as a breast milk substitute for infants under 1 year of age due to its inappropriate nutritional composition.

##### 3.3.1.4 Core deviations between practice and guidelines

In summary, the core deviations between traditional feeding practices for Tibetan infants and young children and scientific guidelines are mainly reflected in the following points: ① Timing of complementary food introduction: Foods are introduced significantly earlier than the “around 6 months of age” recommended by guidelines. ② First complementary food: Traditional zanba paste is used instead of the guideline-recommended “iron-rich foods” (such as iron-fortified infant cereal). ③ Dietary diversity: There is a long-term reliance on a few traditional infant feeding items, with a failure to timely and adequately introduce animal-source foods, particularly eggs and meat, as well as vegetables and fruits. Together, these deviations represent important feeding-related patterns that may be associated with the high burden of malnutrition among infants and young children in this region.

#### 3.3.2 Socio-ecological factors influencing feeding behaviors and associated health outcomes

This review adopts a socio-ecological model to categorize the numerous factors affecting the feeding behaviors of Tibetan infants and young children into three interrelated levels: the individual and interpersonal level, the community and organizational level, and the macro-policy and environmental level. These factors interact to shape current feeding patterns and may be associated, either directly or indirectly, with the severe nutritional and health challenges faced by infants and young children in the region.

##### 3.3.2.1 Individual and interpersonal level

At this core level, knowledge, beliefs, and the economic situation within the family represent proximal factors that may shape feeding behaviors.

*Caregiver knowledge and beliefs*: There is a widespread lack of scientific feeding knowledge among mothers and other primary caregivers. One study showed that only 21.8% of mothers knew that complementary foods should be introduced between 4 and 6 months of age (30). The source of knowledge relies heavily on the experience of elders (39.74%), rather than on scientific guidance (14). Concurrently, traditional beliefs are deeply entrenched; for example, many mothers believe that newborns should be fed zanba paste right after birth to make them “grow strong,” or that breast milk alone is insufficient, necessitating the early introduction of staple foods (43).
*Family structure and social support*: In feeding decisions, grandparents and other elders often have a significant influence, and their traditional experiences are typically prioritized. Furthermore, communication among family members has been reported as a key factor influencing adherence to interventions such as the use of nutrition supplement packages (“Ying Yang Bao”) (40).
*Socioeconomic status*: The mother’s level of education is a significant influencing factor. In many areas, mothers are illiterate, which directly limits their ability to acquire and understand scientific knowledge (14, 32). Family economic status also plays a key role. Research indicates that low per capita monthly household income is a risk factor for childhood anemia (26), and family economic status is also an important factor influencing breastfeeding behavior (19).
*Associated health impacts*: These individual and interpersonal factors are reported to be linked to improper feeding practices, such as low rates of exclusive breastfeeding and the premature introduction of non-diversified complementary foods (14, 19). These practices have been associated with a range of adverse health outcomes. For example, the premature introduction of Tsampa (roasted barley flour) paste is not only reported as a common risk factor for low body weight and stunting in children (28), but it may also be associated with anemia in infants and young children (44). In extreme cases, it has even been considered a possible contributing factor to neonatal necrotizing enterocolitis (NEC) (41). Meanwhile, the prevalence of anemia is significantly higher in mixed-fed and formula-fed infants compared to those who are exclusively breastfed (34).

##### 3.3.2.2 Community and organizational level

*Community culture and social norms*: Introducing zanba as an infant’s first complementary food is not merely a family choice but a widely accepted and practiced community cultural norm (43). Additionally, the dietary habit of drinking “horse tea” (which can inhibit iron absorption) in some areas may also exacerbate the problem of anemia at the community level (21).
*Accessibility and quality of health services*: There is a clear inadequacy of health education support at the organizational level. Existing evidence indicates a common lack of targeted health education materials in the local language (Tibetan) (14). At the same time, the level of trust in community health personnel is a crucial factor influencing whether families adopt nutritional interventions (such as using nutrition supplement packages), which highlights the core role, and potential shortcomings, of primary health services in health communication (40).
*Associated health impacts*: Community-level cultural norms may help sustain non-recommended feeding practices. Meanwhile, the absence of health education at the health organization level may make it more difficult to correct these beliefs and behaviors effectively. This lack of support may be related to the sustained high prevalence of malnutrition problems, such as widespread anemia (7, 34), stunting (6, 32), and micronutrient deficiencies, including the persistent high incidence of rickets due to vitamin D deficiency (23).

##### 3.3.2.3 Macro-policy and environmental level

Geography and Natural Environment: High altitude is an unavoidable macro-level factor affecting the health of Tibetan children. Multiple studies have confirmed that altitude is significantly correlated with malnutrition and micronutrient deficiencies. The higher the altitude, the greater the risk of stunting, underweight (6), and deficiencies in vitamins A and D (37, 45). At the same time, the harsh climate and inconvenient transportation in high-altitude areas make it difficult to obtain fresh foods like vegetables and fruits, which limits dietary diversity (43).

Socioeconomic and Policy Environment: Poverty is an important structural factor associated with malnutrition. At the national level, among all contiguous destitute areas, the Tibetan areas of the four provinces have the most prominent issues with infant and child anemia (66.2%) and stunting (15.9%), rates that are far above the average (22). Furthermore, household sanitary conditions (such as access to safe drinking water and sanitary toilets) are also significantly correlated with anemia and stunting (36). At the policy level, the content of the reviewed studies suggests a potential lack of sufficiently robust food fortification policies or nutrition improvement programs tailored to the cultural characteristics of the region.

*Associated health outcomes*: These macro-level factors are considered important contextual factors associated with nutritional problems. The geographical environment may influence children’s growth, development, and nutritional metabolism. Meanwhile, widespread poverty and relatively lagging public health infrastructure may limit the ability and willingness of families to improve feeding practices, and are associated with the high prevalence of nutritional problems in the region, such as stunting, anemia, and micronutrient deficiencies among children.

## 4 Discussion

### 4.1 Comprehensive interpretation of the results

The findings of this integrative review suggest that feeding practices among Tibetan infants and young children should be understood not as isolated individual choices, but as culturally embedded behaviors shaped by interacting family, community, health-system, socioeconomic, and environmental conditions. This interpretation is theoretically important because it shifts the focus from correcting mothers’ knowledge alone to understanding feeding behavior as part of a broader socio-ecological system. In this system, early introduction of tsampa, low exclusive breastfeeding rates, and limited dietary diversity appear to be maintained by a combination of caregiver beliefs, intergenerational influence, local food availability, limited culturally appropriate health education, poverty, and high-altitude constraints.

At the same time, the quality appraisal findings should temper the interpretation of these results. The evidence is relatively robust for identifying recurring patterns, including early complementary feeding, low exclusive breastfeeding, limited dietary diversity, and the coexistence of anemia and stunting, because these findings were reported across multiple studies. However, because most included studies were cross-sectional and several had limited control for confounding, the strength and direction of specific causal pathways remain uncertain. The qualitative evidence provides valuable cultural explanations for early tsampa feeding, but should be interpreted as contextual and hypothesis-generating. Therefore, the proposed framework should be regarded as an evidence-informed conceptual synthesis rather than a validated causal model.

### 4.2 Theoretical and practical significance of the culturally adapted framework

To translate the complex relationships previously discussed into a preliminary conceptual guide rather than an empirically tested implementation blueprint for policymakers and public health practitioners, this study constructs a conceptual framework for culturally adapted interventions, grounded in the socio-ecological model and the synthesized evidence of this review (see Figure 1). This framework provides a systematic analysis of the problem’s potential multi-level determinants and may offer a structured reference for identifying possible intervention entry points, rather than a tested implementation pathway.

The framework was generated through an evidence-to-framework logic, but the Discussion focuses on its theoretical meaning and practical use rather than repeating the full construction process. As a preliminary conceptual framework, it should be understood as evidence-informed and theoretically triangulated, rather than externally validated. Its practical feasibility, cultural acceptability, and pathway-specific validity require further testing in real-world Tibetan community settings.

The practical significance of this conceptual framework lies in its ability to function as a preliminary diagnostic and planning tool. As a diagnostic tool, it can help local public health agencies identify which ecological levels have been insufficiently addressed. As a planning guide, it highlights the need for multi-sectoral collaboration across health, agriculture, social security, education, and communication sectors. As a conceptual action framework, it links culturally adapted strategies to different ecological levels, emphasizing that individual education, family support, community engagement, health-system strengthening, and supportive policy environments should be considered together rather than as isolated interventions. However, because the framework has not yet been empirically evaluated, its use in practice should be regarded as hypothesis-generating and should be preceded by local feasibility assessment, stakeholder consultation, and pilot testing.

The theoretical contribution of this framework is not the replacement of SEM or SDH, but the operationalization of these theories in a culturally embedded nutrition context. SEM identifies where determinants are located, SDH explains why inequities are structurally produced, and Cultural Translation specifies how biomedical nutrition recommendations can be reformulated into culturally acceptable intervention strategies. For example, the intervention logic is not simply to prohibit tsampa, but to delay its inappropriate early introduction, improve its nutrient density when used at an appropriate age, and communicate this message through trusted family and community channels. In this sense, Cultural Translation functions as a practical mechanism that connects multi-level determinant analysis with culturally embedded intervention design.

Figure 1 illustrates the proposed pathway linking determinants, intervention components, and expected outcomes. Multi-level socio-ecological determinants, including caregiver beliefs, family influence, community norms, health-service accessibility, poverty, food availability, and high-altitude constraints, shape direct feeding behaviors such as early introduction of tsampa, low exclusive breastfeeding, and limited dietary diversity. These feeding behaviors are then linked to adverse nutritional outcomes, including stunting, anemia, and micronutrient deficiencies. The intervention components are designed to act on these determinants and behaviors through Cultural Translation, which reformulates biomedical nutrition recommendations into culturally acceptable strategies at the individual/family, community, health-system, and macro-policy levels. The expected pathway is therefore: culturally translated multi-level interventions → improved feeding practices and food environments → improved nutritional status among Tibetan infants and young children.

### 4.3 Culturally adapted intervention strategies

Based on the socio-ecological analysis, this section outlines four practical entry points for culturally adapted intervention rather than an exhaustive implementation plan. These strategies should be interpreted as theoretically derived intervention options rather than evidence-confirmed practice recommendations. The emphasis is on how the proposed framework can guide intervention thinking at the individual/family, community, health-system, and macro-policy levels, while ensuring that strategies remain culturally acceptable and practically sustainable. Their feasibility, acceptability, and effectiveness require further assessment through stakeholder engagement and pilot implementation in Tibetan community settings. To strengthen the practical grounding of these theoretically derived strategies, the following discussion also draws on comparable examples already reported in the literature, including community-based maternal support and health education, nutrition supplementation programs such as Ying Yang Bao, the Positive Deviance/Hearth approach, and the engagement of trusted community or religious leaders. These examples are used as implementation analogs rather than direct evidence that the proposed Tibetan framework has been validated, indicating that the proposed strategies are culturally adapted extensions of intervention principles used in comparable low-resource, community-based, or culturally embedded nutrition settings.

#### 4.3.1 Individual and family level: implementing “culturally translated” health education

At the core level that directly influences feeding behaviors, the focus of intervention should shift from simple “knowledge transmission” to “cultural negotiation and modification”.

(1) Develop “Culturally Translated” Health Education Materials: Traditional health education often directly negates local practices, which can easily provoke resistance. A more effective approach is to acknowledge and respect tradition before proposing modifications. For instance, the intervention message should not be, “Do not feed infants tsampa,” but rather, “How can we make tsampa more nutritious?” Bilingual (Tibetan-Mandarin) illustrated manuals or short videos could be produced to demonstrate how to enrich traditional tsampa paste with nutrient-dense, locally acceptable ingredients, with priority given to age-appropriate animal-source foods such as eggs, minced meat, or other locally acceptable meat products, because these foods provide high-quality protein and more bioavailable iron and zinc. Vegetables and fruits should also be encouraged as complementary sources of vitamins and dietary diversity, thereby transforming it from a simple energy source into a composite complementary food with higher nutrient density. This strategy reflects the principle of Cultural Translation: traditional infant feeding items are not simply rejected, but are modified and repositioned within age-appropriate, nutrient-dense complementary feeding. It both respects the traditional belief that “tsampa makes children strong” (43) and skillfully introduces scientific knowledge about dietary diversity. This intervention logic is consistent with the Positive Deviance/Hearth approach, which identifies locally feasible feeding and care practices from families in the same community who achieve better child nutrition despite similar resource constraints. This provides a useful precedent for the present framework because it shows how culturally familiar foods and practices can be adapted into more nutrient-dense and age-appropriate feeding behaviors without directly rejecting community traditions.
(2) Conduct Family-Centered Counseling and Empowerment: Feeding decisions are not made by the mother alone; grandmothers and other elders wield considerable influence (14). Therefore, interventions should target the family as a unit. For example, during routine child health services, core family members (including fathers and grandmothers) should be invited to participate in nutritional counseling together to promote intergenerational communication and knowledge sharing. Research suggests that effective communication among family members is key to improving adherence to intervention programs, such as those involving nutrition supplements (40). Through joint family decision-making, feeding behaviors may be more effectively supported and modified. Similar experiences from nutrition supplement programs indicate that caregiver acceptance is influenced not only by knowledge, but also by family communication and support, suggesting that family-centered counseling is a practical component of culturally adapted nutrition intervention.

#### 4.3.2 Community level: fostering social support networks and empowering key individuals

The community is the setting in which feeding norms are formed, reinforced, and transmitted across generations. Therefore, community-level intervention should not only deliver nutrition information, but also reshape the social environment in which feeding decisions are made. For Tibetan families, practices such as early introduction of tsampa are not merely individual choices; they are embedded in shared cultural expectations and intergenerational experience. Thus, community-based strategies are needed to create supportive norms for recommended feeding practices.

(1) Establish “Mother Support Groups”: Mother support groups can serve as a culturally acceptable platform for peer learning, emotional support, and practical problem-solving. These groups may be organized by local health workers or experienced “role-model mothers” who have successfully adopted recommended feeding practices. Through regular group activities, mothers can share feeding experiences, discuss difficulties, and learn practical skills such as how to continue breastfeeding, delay inappropriate early complementary feeding, and improve the nutrient density and diversity of complementary foods. Compared with one-way health education, peer support may be more acceptable because it allows mothers to learn from trusted community members who face similar cultural and economic conditions. Previous evidence suggests that group-based community health education can improve maternal and newborn health behaviors, including early initiation of breastfeeding and exclusive breastfeeding (49, 50). Therefore, mother support groups may help transform scientific feeding recommendations into locally understandable and socially supported practices. These programs therefore provide a relevant best-practice model for Tibetan communities by emphasizing repeated peer contact and locally trusted communication rather than one-time didactic education.
(2) Empower Key Community Figures: In Tibetan communities, respected figures such as religious leaders, village elders, community representatives, and trusted local health workers may strongly influence family beliefs and collective norms. Engaging these figures in nutrition promotion can enhance the credibility and acceptability of intervention messages. For example, rather than presenting recommended feeding practices as external biomedical instructions, community figures can help frame them in ways that are consistent with local values, such as protecting child well-being, fulfilling family responsibility, and accumulating merit through care for vulnerable children. Evidence from public health practice suggests that collaboration with religious and community leaders can facilitate trust-building and community mobilization (51, 52). However, such engagement should be carefully adapted to local contexts and should avoid instrumentalizing culture or religion; the goal is to support respectful dialog between traditional values and scientific nutrition recommendations. Such examples suggest that, in culturally embedded settings, trusted local actors can function as mediators between biomedical recommendations and community values. This role is consistent with the Cultural Translation mechanism proposed in this framework.

Together, peer support and the involvement of trusted community actors may help transform feeding guidance from external instruction into locally supported social practice.

#### 4.3.3 Health system level: strengthening primary service capacity and cultural competence

The primary health service system is a key bridge between public health policy and family-level feeding practices. In Tibetan-inhabited areas, the effectiveness of nutrition interventions depends not only on whether scientific recommendations are available, but also on whether they can be delivered through accessible, trusted, and culturally competent services. Therefore, health-system strengthening should focus on integrating nutrition support into routine maternal and child health care and improving the capacity of primary health workers to communicate with families in culturally acceptable ways.

(1) Integrate Nutrition Services into Routine Maternal and Child Health (MCH) Care: Infant and young child nutrition assessment should be incorporated into routine services at township health centers, village clinics, and community-based child health programs. Such services may include regular monitoring of growth indicators, screening for anemia or micronutrient deficiencies where feasible, assessment of breastfeeding and complementary feeding practices, and individualized feeding counseling. Rather than treating nutrition education as a one-time activity, primary health services should provide continuous, age-specific guidance across the first 1,000 days of life. In addition, stable access to essential nutrition interventions, such as vitamin D supplementation, micronutrient powders, or infant nutrition packs, should be strengthened where appropriate. Previous evidence suggests that trust in community health workers is an important factor influencing whether families accept nutrition recommendations or intervention programs (40). Therefore, improving the professional capacity, continuity, and credibility of primary health services is central to translating the framework into practice. This service-based delivery logic is consistent with experiences from nutrition pack programs such as Ying Yang Bao.
(2) Enhance “Cultural Competence” Training for Health Personnel: Cultural competence is essential for ensuring that feeding guidance is not perceived as external, judgmental, or dismissive of local traditions. Primary health workers should be trained to understand common Tibetan infant feeding customs, family decision-making patterns, local dietary preferences, language needs, and cultural or religious sensitivities. Such training should help health personnel move from simple knowledge transmission to respectful cultural negotiation. For example, health workers can use culturally respectful counseling to explain age-appropriate complementary feeding and practical ways to improve dietary diversity within local food traditions. Culturally competent communication may improve families’ willingness to discuss feeding practices honestly, increase trust in health advice, and reduce the risk that interventions fail because of cultural misunderstanding or resistance (53).

Overall, health-system-level intervention should provide the professional and institutional support needed to sustain family- and community-level behavior change. By embedding culturally sensitive nutrition counseling into routine MCH services, the framework can move beyond episodic health education and toward continuous, relationship-based support for Tibetan caregivers.

#### 4.3.4 Macro-policy and social environment level: creating a supportive policy and food environment

At the macro-policy and social-environment level, culturally adapted health education must be supported by broader structural conditions. Families cannot easily change feeding practices if nutrient-dense foods are unaffordable, unavailable, or inconsistent with local food systems. Therefore, the framework emphasizes that nutrition improvement in Tibetan-inhabited areas should not rely solely on individual counseling, but should be supported by food policy, social assistance, and improvements in food accessibility.

(1) Explore Food Fortification Strategies for Locally Consumed Staples: Given the high burden of anemia and micronutrient deficiencies, food fortification may be an important policy direction. Evidence from low- and middle-income countries suggests that mandatory fortification of staple cereals can reduce anemia among women of reproductive age (54). In Tibetan areas, this principle could be explored in relation to locally consumed staple foods, such as highland barley flour or other cereal products used in the preparation of tsampa. However, such policies should not be presented as ready-made solutions. Their feasibility would depend on local production systems, processing methods, regulatory capacity, cost, community acceptance, and whether fortification can be implemented without undermining the cultural meaning of traditional infant feeding items. Therefore, staple-food fortification can be viewed as a macro-level best-practice analog, but its application in Tibetan areas would require local feasibility assessment, pilot testing, and careful cultural and technical adaptation rather than direct transfer.
(2) Strengthen Social Security and Nutrition Assistance for Vulnerable Families: Poverty and household food insecurity are important structural factors associated with child malnutrition (26, 32). For families living in remote or economically disadvantaged Tibetan areas, knowledge alone may not be sufficient to change feeding practices if diverse foods or nutrition supplements remain unaffordable. Therefore, nutrition interventions should be linked with social security, rural revitalization, poverty alleviation, and maternal-child health programs. Targeted nutrition assistance, such as subsidized micronutrient supplements, nutrition packs, or food vouchers for vulnerable households, may help reduce the gap between knowing what to feed and being able to provide it. Existing experience with nutrition supplement programs, such as “Ying Yang Bao,” suggests that such interventions may be more acceptable and sustainable when integrated with routine child health services and supported by trusted local health workers (40, 55). This example is particularly relevant because it demonstrates that nutrition assistance programs should address both material access and behavioral adoption. For Tibetan-inhabited areas, similar programs would need to combine subsidies or nutrition products with culturally appropriate counseling, family engagement, and trusted local delivery systems.
(3) Improve Food Supply Chains and Accessibility: At the macro-policy level, improving the food environment is essential because culturally adapted counseling alone cannot change feeding practices when nutrient-dense foods remain unavailable or unaffordable. Policies should therefore support both short-term supply mechanisms for nutrient-dense foods, especially age-appropriate animal-source foods such as eggs and meat, as well as fortified foods and fresh produce, and longer-term local food-production capacity adapted to high-altitude conditions. These measures may include improved logistics, targeted subsidies, cold-chain support, locally feasible agricultural technologies, and cooperative food-access models that improve access to nutrient-rich foods while supporting local livelihoods and community ownership. In this sense, food-system resilience functions as an enabling condition for dietary diversity and sustained nutrition improvement in remote Tibetan communities.

### 4.4 Potential global health implications and transferable principles

The nutritional challenges faced by Tibetan infants and young children, although unique in their specific context, are not an isolated phenomenon. Instead, they reflect a broader global health dilemma: the tension between deeply entrenched traditional lifestyles and evidence-based public health guidelines. This tension is also relevant to many indigenous peoples, ethnic minorities, and remote communities worldwide. However, because the evidence synthesized in this review was derived entirely from Tibetan populations in China, the proposed framework should not be regarded as a directly generalizable model. Rather, it should be understood as a context-derived conceptual framework that may provide useful sensitizing concepts for examining similar tensions between tradition and nutrition science in other settings. Its relevance and applicability in other cultural and geographic contexts require further empirical validation.

The core issues identified in this review resonate with findings from different geographical contexts. In many regions globally, complementary foods made from locally available cereals and root crops often have low protein quality, high viscosity, and reduced nutrient density when diluted for infant feeding (56). The reliance on tsampa in Tibetan areas can therefore be viewed as a specific manifestation of a broader pattern. Comparable examples include fermented maize or sorghum porridges such as ogi or pap in sub-Saharan Africa, which may lose essential nutrients during traditional processing and often lack sufficient iron, zinc, and vitamin A (56, 57). In the Andean region, an ethnographic study of Peruvian Quechua communities found that early complementary foods were often carbohydrate-dominant local staples such as potatoes and mazamorra (58). Premastication, an ancient feeding practice (59), has also been described among the indigenous Tsimane people in the Bolivian Amazon, where caregivers use this method to prepare meat, fish, plantains, and other family foods for infants (60). These examples suggest that reliance on culturally familiar but nutritionally limited complementary foods may be a recurring concern in some settings. However, the specific cultural meanings, food systems, and intervention pathways are likely to vary substantially across populations.

Similarly, the multi-level determinants identified through the socio-ecological model may have relevance beyond the Tibetan context. The socio-ecological model has been widely used in child malnutrition research and highlights the importance of determinants at individual, family, community, and macro-environmental levels (61). High-altitude environments, for example, may affect child nutrition through both physiological stress and constraints on agricultural production and food availability, as observed in regions such as the Ethiopian Plateau and the Andes Mountains (58, 62). More broadly, social determinants such as maternal education, household poverty, and poor sanitation have also been repeatedly associated with child malnutrition across low- and middle-income countries (63, 64). Therefore, the culturally integrated intervention framework proposed in this study should be viewed as potentially transferable at the level of broad principles, such as systems thinking, cultural negotiation, and multi-sectoral collaboration, rather than as a universally applicable intervention model.

(1) A Shift to Systems Thinking: The framework emphasizes the need to move beyond singular, linear interventions such as health education and toward multi-level, multi-sectoral approaches. Fragmented interventions may have limited and unsustainable effects on complex health problems, whereas systems-oriented approaches that address policy, community norms, and individual capacity simultaneously are more likely to create lasting change (65). In this sense, the proposed framework provides a structured way to organize culturally embedded nutrition interventions.
(2) From Cultural Replacement to “Cultural Translation”: A key implication of the framework is the shift from “cultural replacement” to “Cultural Translation.” The strategy of asking “How can tsampa be made more nutritious?” rather than simply saying “Do not feed tsampa” represents a more culturally acceptable intervention logic. This principle is consistent with the Positive Deviance/Hearth model, which identifies locally feasible solutions from families within the same community who achieve better child nutrition despite similar resource constraints (66). By transforming local wisdom into practical strategies, this approach may be more acceptable than prohibitive education that negates long-standing traditions. However, whether similar strategies could be used for foods such as traditional fish soup in Pacific Island nations or maize porridge in African settings would depend on local dietary practices, caregiver beliefs, food availability, and health-system delivery channels.
(3) Empowering Key Community Figures: Collaborating with local key figures, such as community elders, traditional healers, and religious leaders, is another important strategy for improving the credibility and acceptance of health interventions. The World Health Organization has emphasized engagement with religious leaders and faith-based organizations as a strategy for community mobilization and trust-building during health emergencies (67). Evidence from COVID-19 vaccine promotion also suggests that the participation of religious leaders can improve vaccine acceptance (68). In the context of culturally sensitive nutrition interventions, such community figures should therefore be engaged as partners who help translate public health recommendations into locally meaningful messages.

In summary, this study, using the Tibetan region as an example, suggests a possible direction in global health practice: a shift from a top-down, unidirectional knowledge output model to a bidirectional, collaborative cultural negotiation model. The significance of the proposed framework lies primarily in its detailed analysis of the Tibetan case and in its potential to inform hypothesis generation and context-sensitive intervention design in other settings. It should not be interpreted as a ready-to-use global blueprint. Future studies are needed to examine whether, how, and under what conditions the framework can be adapted and validated in other cultural and geographic contexts.

## 5 Conclusion

This integrative review shows that infant and young child feeding practices among Tibetan populations are culturally embedded behaviors shaped by a multi-level socio-ecological system. The main feeding-related problems include low exclusive breastfeeding rates and the premature, non-diversified introduction of traditional staple-based complementary foods, particularly Tsampa, which may be associated with stunting, anemia, and micronutrient deficiencies among Tibetan infants and young children.

The main contribution of this study is the development of a culturally adapted conceptual framework that links socio-ecological determinants with intervention design. By integrating the socio-ecological model with Cultural Translation, the framework highlights that improving Tibetan infant nutrition requires more than health education alone; it requires culturally acceptable, multi-level, and multi-sectoral strategies that address family beliefs, community norms, health-service capacity, food accessibility, poverty, and high-altitude constraints. The framework may serve as a preliminary planning reference for Tibetan-inhabited areas and offer conceptual insights for culturally embedded nutrition interventions in other settings, although its transferability requires empirical validation in different cultural and geographic contexts.

## 6 Limitations and implications for future research

Despite providing a comprehensive perspective on infant and young child feeding issues in the Tibetan population, this integrative review has several limitations. First, the vast majority of the literature included in this analysis were cross-sectional studies (30 out of 35 papers). While this design can reveal associations, it has limited capability in establishing causal relationships between feeding practices and health outcomes. Therefore, many of the “influencing factors” and “determinants” identified in the “Results” and “Discussion” sections of this review are, to a large extent, inferences based on correlational evidence. The causal pathways and their strength between these factors and malnutrition still await confirmation from future higher-quality evidence, such as cohort studies or intervention trials. Second, heterogeneity exists across the included studies in terms of geographic coverage (despite being broad, gaps remain), sample size, and measurement methods. This may affect the generalizability of the results.

Moreover, because all included studies focused on Tibetan populations in China, the framework is grounded in a highly specific cultural, geographic, dietary, and health-system context. A further important limitation is that the proposed framework remains theoretical and has not yet been evaluated in practice. Although it was developed through evidence synthesis, internal consistency checking, and theoretical triangulation, no intervention study, pilot program, stakeholder consultation, or community-based validation has been conducted to assess whether the proposed pathways and strategies are feasible, acceptable, or effective in real-world Tibetan settings. Therefore, the framework should be interpreted as a conceptual model for guiding future intervention development rather than as a validated practice model. Claims regarding its applicability to other indigenous, ethnic minority, or remote populations should therefore be made cautiously. The framework may offer transferable concepts, but its relevance, acceptability, and effectiveness in other settings remain uncertain and require empirical validation.

The quality appraisal further indicated several methodological weaknesses that may influence confidence in the synthesized findings. Some quantitative studies did not clearly report participant recruitment procedures, sample representativeness, or adjustment for important confounding factors. The limited number of longitudinal and qualitative studies also restricted the depth of causal and cultural interpretation. In particular, although the qualitative study provided valuable insight into local feeding beliefs, limitations in reporting reflexivity, ethical considerations, and analytic rigor reduce confidence in the completeness and transferability of these cultural explanations. Therefore, the conceptual framework should be interpreted as a preliminary synthesis of the best available evidence rather than a framework supported by uniformly high-quality empirical studies.

Finally, as an integrative review, this study is dependent on previously published literature, suggesting a potential for publication bias, where studies reporting positive or significant findings are more likely to be published. In addition, this study only retrieved Chinese and English literature, and may have omitted relevant materials or reports published in local languages such as Tibetan. Finally, and most importantly, the intervention framework constructed in this study (Figure 1) and the proposed intervention strategies (Section 4.3) are theoretical constructions based on existing evidence and the authors’ integrative interpretation of the reviewed studies. Although internal consistency checking and theoretical triangulation were conducted during framework development, no formal external validation was performed through expert consultation, Delphi consensus, stakeholder review, or direct input from Tibetan caregivers, community health workers, religious or community leaders, and local policymakers. Therefore, the practical feasibility, cultural acceptability, cost-effectiveness, and real-world intervention effects of this framework and its related strategies remain to be empirically tested in future research.

Building on the limitations of the current evidence, future research should focus on the following directions to generate higher-quality evidence:

(1) Conduct Large-Scale Prospective Cohort Studies: This is the most urgent need. There is an imperative to design and implement large-scale, multi-center cohort studies that longitudinally follow Tibetan mother-infant dyads, beginning in pregnancy. These studies should systematically document the dynamic changes in feeding patterns and regularly monitor children’s growth and development, nutritional status, and cognitive development. This would provide stronger longitudinal evidence for examining the potential long-term effects of various feeding practices, particularly traditional staple-based complementary foods, on child health.
(2) Conduct Empirical Evaluations Based on the Intervention Framework: The conceptual framework proposed in this paper provides a preliminary basis for developing and empirically evaluating systematic interventions. Future research should build upon this framework by designing and implementing multi-level, culturally adapted interventions across diverse community settings. Before large-scale intervention testing, future studies should first conduct conceptual and contextual validation of the proposed framework through expert panels, Delphi consultation, stakeholder workshops, and participatory review with Tibetan caregivers, elders, community health workers, religious or community leaders, and local policymakers. Such validation would help assess whether the proposed pathways, intervention targets, and Cultural Translation strategies are understandable, acceptable, and feasible in local practice. In addition, comparative studies in other culturally distinctive and geographically remote populations are needed to examine whether the broad principles of the framework can be adapted beyond the Tibetan context, and to identify which components are context-specific and which may be transferable. This requires employing rigorous designs, such as Randomized Controlled Trials (RCTs) or quasi-experimental designs, to scientifically evaluate the effectiveness, cost-effectiveness, and sustainability of these comprehensive intervention strategies.
(3) Deepen Qualitative and Mixed-Methods Research: More in-depth qualitative research is needed to explore the cultural logic, social motivations, and family dynamics underlying feeding decisions. Examples include exploring the specific roles of grandmothers in feeding decisions and how families negotiate conflicts between traditional practices and modern medical advice. Mixed-methods studies, which integrate qualitative findings with quantitative data, can provide more nuanced and contextually relevant insights for the design of future interventions.

## 7 Implications for public health practice and policy

This review provides preliminary directions for future public health practice and policy planning aimed at improving the nutritional status of Tibetan infants and young children:

(1) Adopt Systemic Thinking to Promote Multi-Sectoral Collaboration: Policymakers and program implementers should move beyond isolated approaches. Improving infant and young child nutrition is not solely the responsibility of the health sector, but requires synergistic policy efforts and collaborative advancement with the Agriculture Sector (to improve the food supply chain and diversity), the Civil Affairs and Poverty Alleviation Sectors (to implement social security and nutrition assistance programs), the Education Sector (to enhance female education levels), and the Publicity/Communication Sector (to disseminate scientific knowledge through local media).
(2) Position “Cultural Adaptability” as the Core Principle of Intervention Design: Future interventions should respect and be embedded within the local cultural context. In practice, strategies of “cultural translation” or “cultural grafting” should be incorporated into intervention design, i.e., proposing modified solutions while acknowledging and respecting traditional beliefs (e.g., “How to make tsampa more nutritious?”). Concurrently, empowering key figures such as community leaders and religious figures to become disseminators of health knowledge may help improve the acceptability of future interventions.
(3) Strengthen the Capacity Building of the Primary Health Service System: Primary health workers represent the “last mile” connecting policy to families, and their role is crucial. Infant and young child nutrition counseling and screening should be fully integrated into routine Maternal and Child Health (MCH) services. Furthermore, training for primary health workers in “cultural competence” should be intensified, enabling them to provide scientific guidance in a manner that is both understandable and acceptable to the local population.
(4) Increase Investment in a Supportive Macro-Environment: The government should proactively explore and promote targeted macro-policies, for example, investigating the feasibility and legislation for nutrient fortification in highland barley flour (qingke mian). This also includes expanding the coverage of social assistance programs like the “Ying Yang Bao” (Nutrition Pack), to create a conducive external environment for improved individual behavior.

Ultimately, we call upon policymakers and researchers to dedicate more attention and resources to this field, using the conceptual framework proposed in this study as a preliminary planning reference to be refined through stakeholder consultation, pilot testing, and empirical evaluation, to collectively commit to eliminating health inequalities and ensuring that every Tibetan child has a healthy and equitable start to life.
